# Optimizing Clinical Study Report Development Through Lean Authoring: A Framework for Meeting Regulatory Expectations and Preparing for Automation

**DOI:** 10.1007/s43441-026-00980-6

**Published:** 2026-06-10

**Authors:** Lauren Geary, Inger Ødum Nielsen, Fiona Charron, Joanne J. Carter, Louise Martinsson, Eve Rowe, Aimalohi Goese, Animesh Chaturvedi, Madhavi Gidh-Jain, Stephanie Hreha-Phillips, John April

**Affiliations:** 1 Eli Lilly and Company, 893 S Delaware St, Indianapolis, IN 46225 USA; 2 Novo Nordisk A/S, Copenhagen, Denmark; 3 Sanofi, Paris, France; 4 F. Hoffmann-La Roche Ltd, Basel, Switzerland; 5 GSK Plc, London, UK; 6 Pfizer Inc., Tadworth, UK; 7 Novartis Healthcare Pvt Ltd, Hyderabad, India; 8 Merck and Co., Inc. (MSD Outside of the USA), Kenilworth, USA

**Keywords:** Lean authoring, Clinical study reports (CSRs), Regulatory documents, Clinical documents, Automation, Digital transformation

## Abstract

This review article provides a comprehensive overview of lean authoring in the pharmaceutical industry based on the experience of medical writing leaders, emphasizing its importance for efficiently creating clinical study reports (CSRs) and other clinical documents. Lean authoring is defined as an approach focused on delivering unambiguous, clear, concise, and objective content that meets regulatory reviewer expectations. This will better ensure accessibility, eliminating unnecessary details, and tailoring content to meet the following needs of regulatory reviewers during what is essentially a non-linear review process: a) fast navigation to decision-critical information, b) a clear, logical trail from study design to analyses to results, c) low cognitive burden, and d) fewer review questions resulting from ambiguous language and inconsistency. The benefits of lean authoring include improved readability and reusability of content, enhanced efficiency and cost-effectiveness in document preparation, and more efficient regulatory reviews. These benefits have been observed across many industries. And yet, challenges in implementing lean authoring methods, including resistance to change, are common. To help companies counter resistance to change, this article outlines the benefits of lean authoring and a step-by-step approach to adopting lean authoring methods, understanding regulatory reviewers’ needs, and developing a robust change management plan. Healthcare agency regulatory reviewers have stated their preference for clear and concise information. Our goal is to influence the pharmaceutical industry to adopt lean authoring to achieve this. By optimizing the authoring process, we aim to increase the efficiency of the process, improve the quality of CSRs, and reduce the burden of regulatory reviews.

**Supplementary Information:**

The online version contains supplementary material available at https://doi.org/10.1007/s43441-026-00980-6.

## Introduction

### Lean Authoring

Lean authoring is an approach to content creation that emphasizes clarity, brevity, and logical flow to effectively communicate key messages [1–3]. In the pharmaceutical industry, various factors enhance this practice, including using International Council for Harmonisation (ICH) guidance and lean templates, integrating new solutions, like artificial intelligence (AI), and meeting the expectations of healthcare regulatory agencies, audiences, and previous applicable regulatory feedback.

Lean documents enable regulatory reviewers to quickly and easily access relevant information and avoid information fatigue [4]. In today’s tech-enabled environment, regulators don’t need repetitive content. Eliminating repetitive content and focusing on key messages enables reviewers to think critically about data without being overwhelmed by it [5]. As a result, lean authoring facilitates efficient and effective healthcare regulatory agency review of clinical documents [6].

### Business Problem

One of the primary challenges encountered in clinical document authoring is tailoring content to the audience: regulatory reviewers. If project teams don’t have a sufficient understanding of their audience, applicable regulations, and the document’s purpose, they might author clinical documents to serve off-target purposes.

Additionally, entrenched academic cultures within clinical development organizations exacerbate natural resistance to change. Such a culture may also have concerns that lean documents don’t include enough information to satisfy regulatory reviewers’ needs. In at least one company, despite efforts to trim unnecessary bulk using an optimized CSR template, CSRs containing over 10,000 pages remained the norm.

To counter these challenges, using CSRs as an example of clinical documentation, this article provides practical guidance on how to implement lean authoring, solutions to overcome challenges to implementation, and regulators’ perspectives on the benefits of lean content.

## Methods

Regulatory medical writing leaders from 8 pharmaceutical companies collaborated to share experiences and practices on adopting lean authoring. We reviewed industry expert knowledge, information publicly available in government websites, journal articles, and professional service vendors’ websites, and created definitions of lean authoring and step-by-step recommendations for its adoption.

## Lean Authoring Principles

The artist, Hans Hofmann, anticipated the lean authoring concept when he stated that simplicity “eliminates the unnecessary so that the necessary may speak [7].” He meant that less is more and that simplicity aids in understanding better than complexity. Simplicity is a design choice that reduces interference with the reader’s purpose of engaging with documentation. Minimalist technical communication treats documentation as a means of supporting action, providing only the information necessary to enable the reader’s understanding [8, 9].

Lean authoring methods follow three high-level principles: clarity, conciseness, and targeting content to the primary audience. Combined, these principles result in content that is easier for the intended reader to navigate and interpret. They eliminate “noise” so that the text can focus on decision-critical information and evidence.

**Fig. 1:**
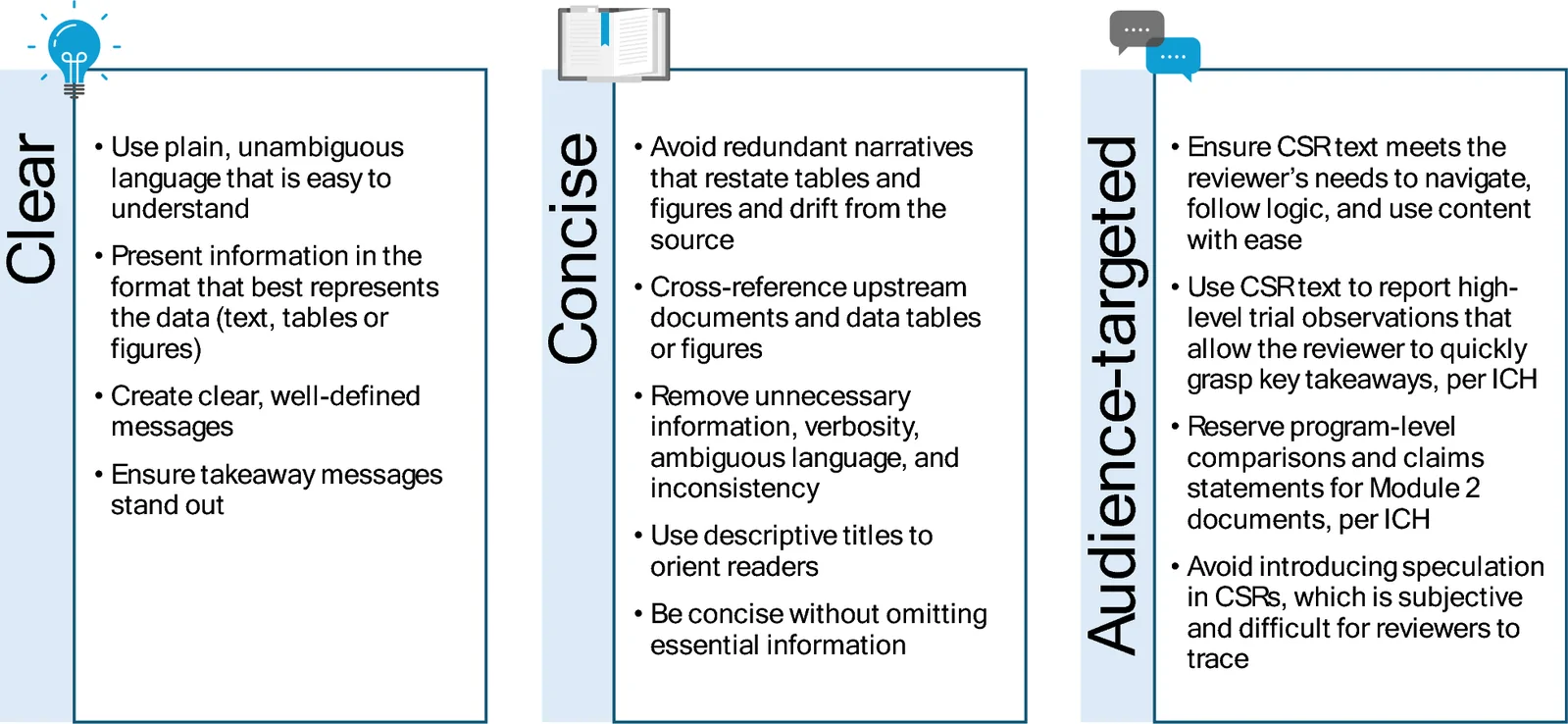
The three principles of lean authoring are clarity, conciseness, and targeting the primary audience. The three principles of lean authoring are each defined. The definitions describe how lean authoring eliminates “noise” in CSRs so that key information stands out.

Figure 1 details how these principles apply to CSRs. Lean CSRs do not mean less scientific rigor. CSRs are meant to report what was observed in a single clinical trial without introducing speculation. As described by ICH, the best way to report clinical trial data is typically in tables and figures. Therefore, a lean CSR must critically evaluate the text’s purpose.

### Writing Principles and Practices Involved in Lean Authoring

Lean authoring methods involve several writing principles and practices. Although the methods in Table 1 are described in the context of CSR generation, many can be broadly applied to clinical document development.

**Table 1:** Lean authoring principles and practices applied to clinical study reports

Principles and practices	Description
*Tools/vehicles*	
Use templates	- Templates enable consistency and efficiency. - TransCelerate protocol and CSR templates effectively complement lean authoring.
Use visual aids as the primary way to convey results	Tables and figures aid comprehension by communicating complex information in an accessible, logical, and orderly format.
*Mechanics*	
Use consistent mechanics and structure	To help readers predict and understand content faster, follow - Grammatical conventions - Standardized formatting and sentence construction, and - Structured authoring rules.
*Methods*	
Reduce noise	- “Create once, use often,” or reference content manually or with digital tools in downstream documents [19]. - Reduce duplicate reporting and results-driven discussions that are not in scope for the CSR per ICH. - Cross-reference to the protocol and statistical analysis plan (SAP) rather than repeating information in the CSR.
*Principles*	
Write to the target audience	- Understand the target audience, their knowledge level, and their objectives. - Prioritize the needs of the target audience over the needs of other audiences, such as internal stakeholders.
Focus text on key messages	- Use text to highlight key messages from the data that are not readily apparent in the tables and figures. - Ensure each sentence has a unique purpose.
*Change management*	
Engage in continuous shared learning	- Peer-review documents to ensure they adhere to lean principles. - Study how others in the industry implement lean authoring methods and tools. - Create training and continuing education programs to ensure employees remain consistent with the change.

### Shifting Mindsets to Implement Lean Authoring

Authoring teams must shift their mindsets to effectively implement lean authoring methods. This section focuses on key cultural shifts to overcome natural resistance to change and increase the chances of successful adoption.

#### From Mere Compliance to Delivering Efficiencies

Adhering to regulations is critically important, but regulatory guidelines often lack prescriptiveness. Change leaders in the industry are shifting their focus toward creating efficiencies for reviewers while still adhering to regulations.

Engaging authoring teams in strategic content planning before creating the first draft reduces rework, prevents duplication and copy-paste drift, and improves alignment on the content approach. Create a topic list with them and define supporting analyses and key conclusions. Educate the authoring team on how writers will adhere to lean authoring principles.

During authoring, assess the usefulness of each content element by questioning whether it helps the reviewer understand and verify the information. Also, assess whether the content is presented in the most appropriate format and location within the broader context of the Electronic Common Technical Document (eCTD). Writing consistently at this level requires strategic content planning.

#### From Author-Centric to Reader-Centric

Authoring team members who are not trained in technical writing may attempt to impose their personal style on CSR writing. An author-centric approach does not support the regulatory reviewer’s task nor the CSR’s purpose. Writers trained in lean authoring should explain the difference between author-centric and reader-centric content, and how the latter helps reviewers evaluate trial data more efficiently. Writers should also explain their expectations of the cross-functional stakeholders on the authoring team. Cross-functional stakeholders contribute their expertise while writers transform this knowledge into lean content that meets the audience’s needs [6].

#### From “More is Better” to “Less is More”

The European Commission has stated that “a clear document will be more effective, and more easily and quickly understood” [2].

Therefore, abandon the belief that large, verbose documents equate to quality [6]. Focus on delivering essential information concisely and clearly. Recognize that documents are not meant to be exhaustive or to contain all information, but that documents exist in a broader context. Each document should have a clear purpose and scope within the broader clinical document ecosystem.

#### From Subjective to Objective

ICH E3 guidelines indicate that CSRs convey a factual summarization of study data. Objective, factual content is transparent and impartial, guarding the CSR from bias and preserving the trial’s scientific integrity [10]. Subjective descriptions do not belong in clinical documents at all. Interpretation and comparisons to other aspects of a clinical program or trials belong in module 2 documents instead of CSRs.

#### From Resisting Change to Embracing Continuous Improvement

Adopt new, efficient authoring methods while challenging traditional practices. Guidelines spanning over 50 years indicate that a wide range of sectors, including aerospace and defense, have found lean authoring principles to be more effective than traditional writing practices [11–13]. By adopting lean authoring, the pharmaceutical industry is starting to see benefits that other industries have long known.

## Applying Lean Authoring Methods to Clinical Study Reports

Table 2 demonstrates the techniques and tools used at the authors’ companies to create leaner, objective, ICH-driven CSRs. As slightly different approaches to these methods exist, we’ve collated experiences from our companies into individual steps that others can follow.

**Table 2:** Initiatives to support objective, lean, ICH-driven clinical study reports

	ICH content ecosystem mapping^1^	Content reuse	Cross-refer, don’t repeat protocol	TransCelerate-driven templates	Structured authoring^2^	Automation in authoring^3^
GSK		In progress	In progress	✓	In progress	In progress
Lilly	In progress	In progress	In progress	✓	✓	In progress
Merck	✓	In progress	✓		✓	In progress
Novartis		In progress	In progress	In progress	In progress	In progress
Novo Nordisk	✓	✓	✓	✓	✓	✓
Pfizer	✓	✓	✓	✓	✓	In progress
Roche	✓	In progress	In progress	✓	✓	✓^4^
Sanofi	✓	✓	✓	✓	✓	In progress

The data in this table reflects current practice at the authors’ companies as of the date of this publication

Abbreviations: ICH, International Council for Harmonisation; Lilly, Eli Lilly and Company

^1^ICH content ecosystem mapping refers to the additional step of mapping ICH E3 guidelines in the context of the rest of the eCTD and ICH modules

^2^Structured authoring at the authors’ companies includes writing rules and component authoring systems. Some companies also leverage TransCelerate’s component content libraries

^3^Automation encompasses some or all of the following: natural language generation, natural language processing, large language models, retrieval-augmented generation, customized coding solutions, software agents, and Amazon Web Services

^4^Available for select clinical study reports

The first steps towards applying lean authoring methods happen at an organizational level, before writing a CSR. In an ideal state, a medical writing organization would begin its content transformation from traditional to lean authoring by identifying its primary audience, mapping its content ecosystem, and generating rules for reuse and de novo content generation.

### Identifying the Primary Audience

The spectrum of potential audiences within the clinical document landscape is diverse and requires tailored communication strategies. The primary audience of CSRs is regulatory agency reviewers who are responsible for ensuring regulatory compliance and reviewing data supporting the conduct of the clinical trial and the results (Supplemental Table 1). These reviewers use CSRs as technical resources and are likely to review them by topic and not from front to back [5, 14]. Authoring teams must remember that this audience differs from internal audiences or those at medical journals.

As part of the audience analysis, consider how regulatory reviewers at the FDA and EMA differ. FDA review committees contain cross-functional experts who rely on their own re-analysis of data. EMA rapporteurs from a diverse range of backgrounds are responsible for creating assessment reports, which collate information across the submission package for EMA’s scientific committees to review [5, 14]. Rapporteurs and EMA reviewers rely on predictably organized, clear, and concise documents to facilitate their ability to find and include key information in their assessment reports.

### ICH-Driven Document Ecosystem Content Mapping

To realize a fit-for-purpose CSR and avoid introducing unnecessary elements, companies should map the broader ICH guidelines that impact documents reporting efficacy and safety results, namely ICH E3, E9, M4E, E2C, and CIOMS III and V. Content maps and their associated rules standardize the content ecosystem, ensuring that cross-functional authoring teams place decision-critical information where regulatory reviewers expect to find it. If content is not in-scope for the CSR, the content map and rules guide authoring teams to place it elsewhere. For example, if an authoring team wants to include in the CSR body individual patient vignettes in addition to patient narratives per ICH E3, they are directed to the guidance for the Summary of Clinical Safety in ICH M4E. Teams wanting to add critical commentary about the interpretation of the study results are directed to the ICH M4E Module 2.5, Clinical Overview. Teams wanting to draw conclusions between laboratory value changes and adverse effects are directed to conducting core safety analyses for the company core data sheet according to ICH E2C (R2) and CIOMS III and V using aggregated data across studies.

Once content is mapped and content rules generated, additional steps to prepare for lean authoring, include

- Defining what content to reuse versus reference to prevent duplication drift
- Identifying the appropriate statistical outputs to include in the report, and
- ensuring that the CSR template instructions direct users to follow lean authoring principles.

### Prepare Upstream Projects with the Downstream Goal in Mind

The next step towards producing a lean CSR should be taken at the time of writing the protocol and SAP.

#### Ensure Content is Reusable

When preparing the protocol and SAP, keep the regulatory reviewers and internal governance readers, as well as downstream reuse, in mind. Protocols and SAPs should use objective, plain, and unambiguous language, well-defined messages, and be concise without omitting essential information. Several companies, including Lilly and Pfizer, use the slogan “say it once, say it well” to educate cross-functional teams on how to envision upstream document development. Writing content well once reduces the need to repeatedly re-engineer content about the same subject, saving time and effort in generating and reviewing clinical documents.

#### Cross-Reference to the Protocol and Statistical Analysis Plan

Reusing the protocol objectives and endpoints of a clinical study in the CSR Synopsis and Study Objectives, Endpoints, and Estimands sections is useful to frame the CSR methods for reviewers. Repetition beyond those anchor locations risks encouraging copy-paste drift and does not improve regulatory reviewer understanding or verification. For example, descriptions of dose adjustments and censoring rules can be cross-referenced to the protocol instead of being repeated in the CSR.

Evidence that regulators favor cross-references to the protocol rather than detailed methods repeated in the CSR was in the December 2010 version of the FDA Manual of Policies and Procedures for Good Review Practice, which had stated that

*Reviewers are expected to use the study/clinical trial protocol for discussions on study/clinical trial design and planned efficacy analyses [i.e. methods] and not the final report itself, because documentation of the study/clinical trial design and the statistical analysis plan within the final report are occasionally incomplete or inaccurate.*

### Streamline the List of Analyses that Support the CSR

The number of objectives, endpoints, and predefined statistical analyses also have downstream implications for the CSR. Presenting only decision-critical data for verifying primary and secondary endpoint conclusions and key safety characterization, per ICH E3 and E9, helps keep the volume of data presented in the CSR manageable, yet sufficient.

For example, analytics, statistics, and writing experts within Lilly, Merck, and Sanofi identified an optimal core set of statistical outputs to include in CSRs that provide agencies with what they need for primary and secondary endpoints and standard safety topics. Additional data are available upon request.

### Adopt the TransCelerate Protocol and CSR Templates

The ICH E3 guidelines released in 1995 intended to “assist sponsors in the development of a report that is complete, free from ambiguity, well organized, and easy to review.” However, they were not intended to be adhered to as a strict template [15]. In more recent years, global agencies, including the FDA, have sought to dispel this interpretation and have encouraged leaner construction, such as through the ICH E3 (R1) Q&A document. Despite this, evidence suggests that sponsors still use ICH E3 as a verbatim template.

In 2015, the cross-pharma consortium TransCelerate BioPharma Inc. harmonized the interpretation of the ICH guidelines. TransCelerate consists of a consortium of pharmaceutical industry peers that partners and consults with representatives from the FDA, EMA, PMDA, and other global healthcare regulatory agencies. The TransCelerate template suite streamlines the creation of protocols and CSRs by providing a leaner, standardized format that aligns with ICH E3 guidelines. These templates are highly encouraged by healthcare regulatory agencies, including the EMA and FDA.

The TransCelerate CSR template emphasizes the need for unique purposes for communication elements. For example, rather than providing a table, figure, and text to describe a result, authoring teams should choose which approach best communicates the study’s findings and simply state in the report body text whether clinically significant changes were observed. This is consistent with ICH E3 (R1).

Furthermore, the TransCelerate template encourages authoring teams to display data in orderly rows and columns rather than in text. Finally, it suggests that sponsors cross-reference the protocol for more granular details about the study design and analytical methods rather than retyping them in the CSR.

The TransCelerate template is most effective when combined with additional rules to guide the content and structure. The process to generate these rules is described under the ICH content mapping and structured authoring subtopics. Accompanying rules can exist as a standalone content guide or be merged into the template itself. Merck, Novo Nordisk, Pfizer, and Roche found merging to be most effective.

### Adopt Structured Authoring Methods

While lean authoring ensures CSR content meets the needs of regulatory reviewers by being unambiguous, clear, concise, and objective, structured authoring provides a writing standard with rules to support reuse and automation.

Structured authoring rules follow cognitive principles that ensure each piece of content is focused on a single topic, each topic begins with a meaningful title, and writers generate topics using consistent terms and sentence structures. These conventions reduce cognitive effort by presenting information in predictable patterns and visually separated sections, which supports efficient reading and comprehension [16, 17]. These rules complement lean authoring by ensuring regulatory reviewers can scan documents and quickly find what they need.

While structured authoring supports HTML- and XML-based information architecture, the writing rules are platform agnostic and can be adopted without involving technology. If companies are not ready for digital architecture, the rules can be used in ordinary word processing software. Vendors are readily available to support companies that wish to adopt structured authoring regardless of the platform.

Lilly based its version of structured authoring methods on Professor Robert E. Horn’s information mapping research [18], the US federal government’s Plain Language Act of 2010, the US federal guidelines of clear and focused writing [17], and the European Commission’s publication titled “*How to write clearly*” [2].

**Fig. 2:**
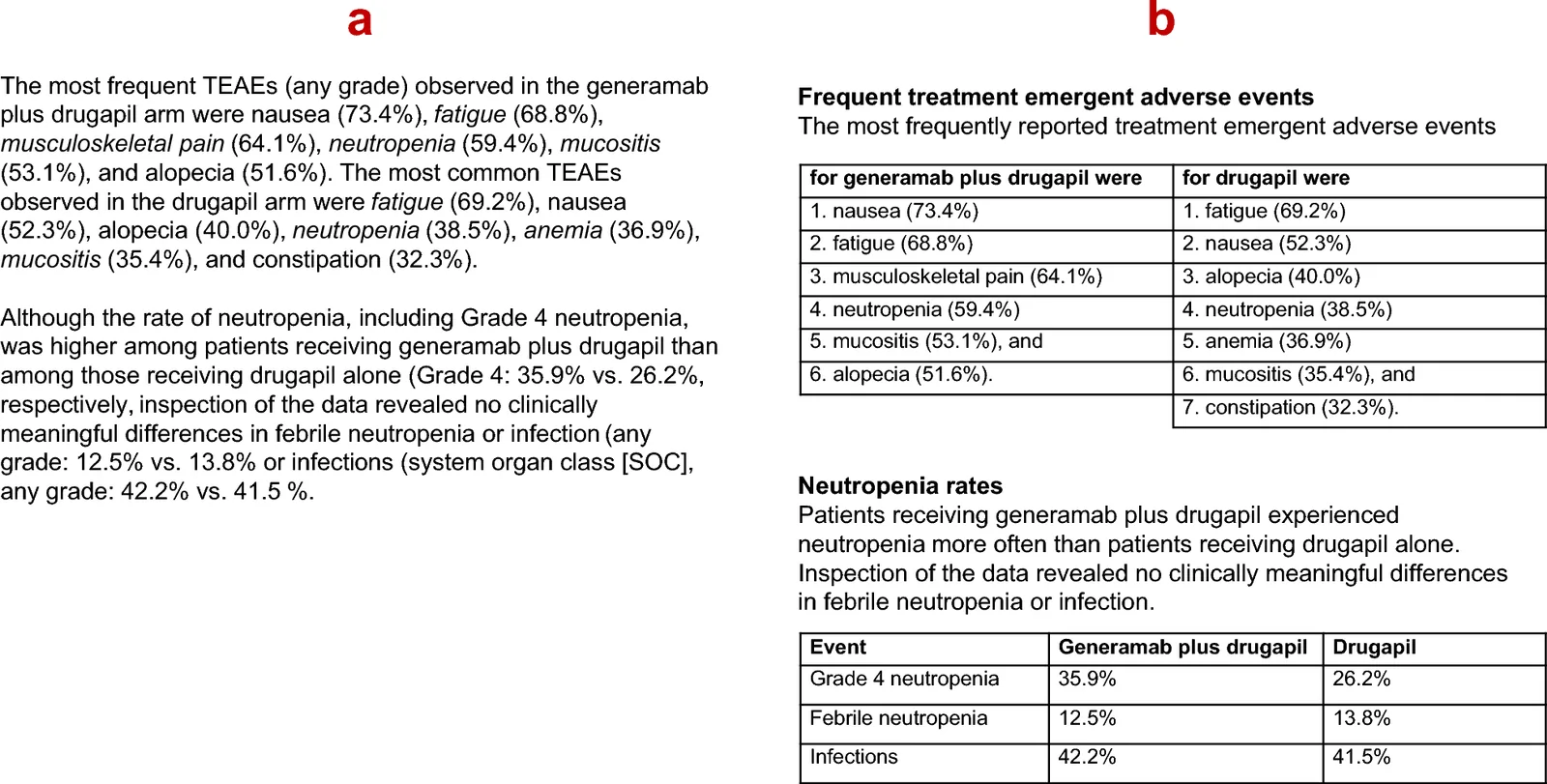
Comparison of unstructured (**a**) and structured (**b**) content in clinical study reports. Figure **a** displays an example of unstructured content. Figure **b** displays an example of structured content. The tables in Figure. **b** show ordered lists of adverse events for generamab plus drugapil and drugapil alone. The tables better organize the data than the text could and provide additional value to the reader by displaying the events in descending frequency.

To avoid redundancy, tables, figures, and text are assigned unique functions to support the reviewer’s task. Tables and figures display data, while text provides commentary and conclusions that are not readily found in visual data displays (Fig. 2).

*Bottom line:* Text must have a unique function to be present in the report.

### Structured Authoring Paired with Component Authoring Systems

In structured authoring, writers organize content into predefined, topic-focused components. Organizing content into small, topic-focused components with unique purposes means they can be easily managed, updated, and reused in different documents and in different contexts [19]. Examples of small, topic-focused protocol components that can be reused in the CSR include objectives, endpoints, and methods. An example of a reusable CSR component is a standard reference statement like “Section [insert reference] contains additional details on this topic.”

When structured authoring is implemented using XML- or HTML-based standards, such as the Darwin Information Typing Architecture, content is not only organized for human readers but is also explicitly encoded with machine-readable structure and metadata. In these environments, content units, including topics, paragraphs, lists, tables, or data elements, are uniquely identified and tagged according to their information type, role, and context. This enables component authoring systems to store, retrieve, and reuse discrete content [16–19].

Beyond identifying content components, structured authoring also improves the semantic organization of text. Sentence-level structure, controlled terminology, and consistent information typing make the functional role of content more explicit. This semantic clarity reduces ambiguity for both human reviewers and downstream systems, and it enables more precise indexing, search, and reuse of content components.

### Structured and Lean Authoring Provide a Foundation for Artificial Intelligence

Lean authoring and structured authoring not only provide many benefits to reviewers, as discussed. They also help AI to automate CSRs.

Subsets of machine learning-based AI, including natural language generation (NLG), natural language processing, and generative AI (GenAI), use deterministic or probabilistic models to understand human language and generate content. Earlier NLG systems relied primarily on deterministic or rule-based approaches to transform structured content into predefined, human-like text. GenAI systems use probabilistic models trained on large corpora of text or curated information to create new content based on learned patterns.

Using GenAI to create clear and concise documents involves training AI models on content that adheres to lean authoring principles. Starting with a lean CSR can make the process of automation easier, as it sets a standard for high-quality content. *In essence, AI is only as good as what it is trained on*.

AI models trained with structured content, that is well-labeled and semantically-defined, can achieve comparable or improved performance with substantially less training data than models trained on larger volumes of unstructured content [20].

On the other hand, poorly structured and verbose text makes relevant information harder for AI systems to identify and isolate.

Novo Nordisk is one of the first pharmaceutical companies to automate CSRs. Prior to doing so, Novo Nordisk adopted a lean and structured CSR template that aligned with the TransCelerate template. In the new template, data-dependent sections relied primarily on references to the TFLs except for specific results sections that provided conclusions from the data. Novo Nordisk enabled CSR automation by creating a digital tool that couples a pre-defined and pre-approved text library, protocol information, selected stakeholder input, and actual study results. The text library was developed by analyzing historical CSRs.

It should come as no surprise that not only are pharmaceutical companies adopting AI, but healthcare regulatory agencies are, as well. The FDA recently announced an agency-wide rollout of GenAI to assist in the scientific review process [21, 22]. Similarly, the EMA is exploring using AI, stating that “AI has the potential to transform the [EMA’s] core work of evaluating human and veterinary medicines for authorisation and monitoring their safety [23, 24].” In other words, if companies embrace the steps we’ve outlined to prepare for AI-driven clinical document development, they will also be better prepared for machines to review their scientific discourse.

## Benefits of Lean Authoring

**Fig. 3:**
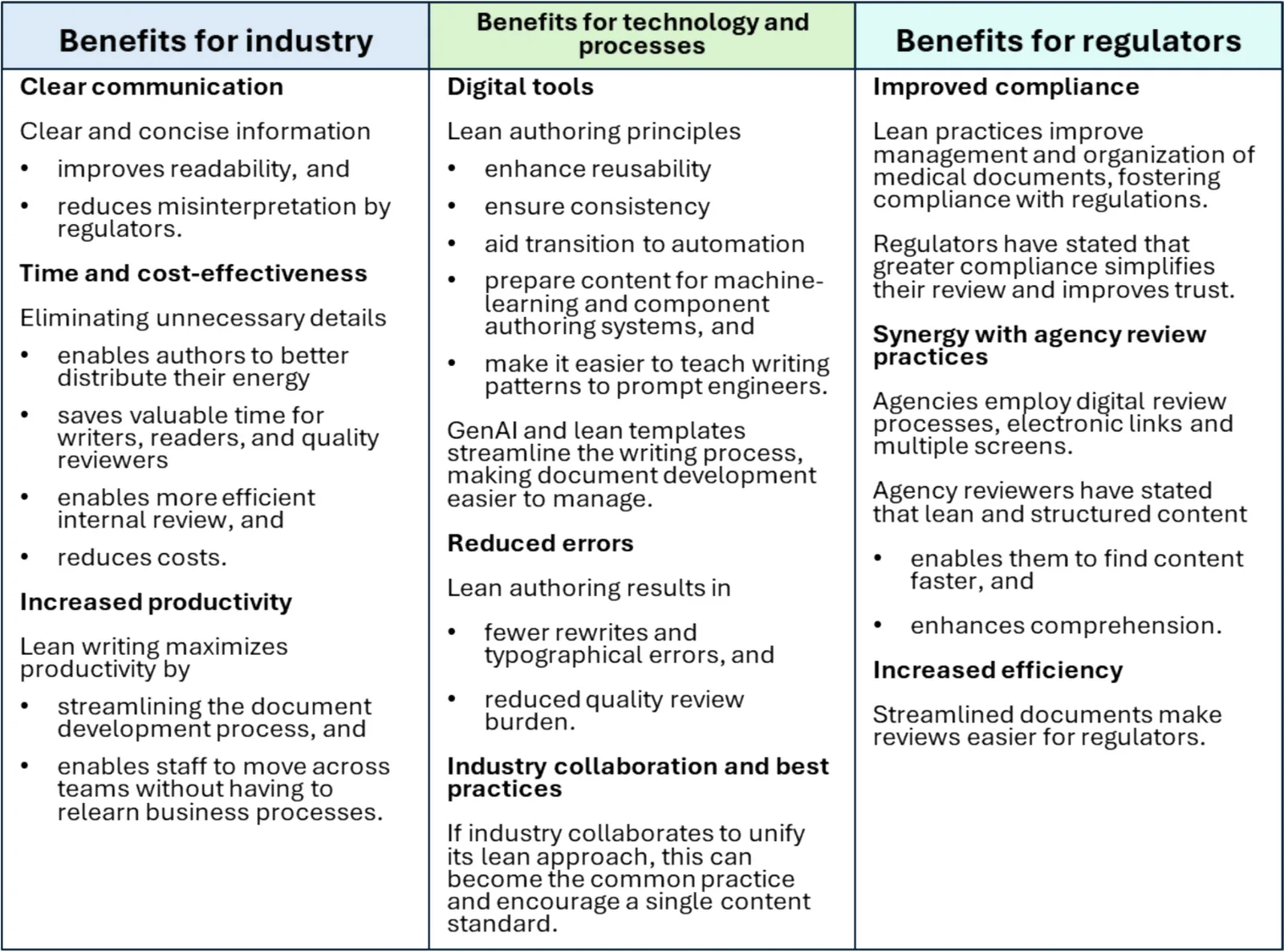
Benefits of lean authoring. The benefits of following lean authoring principles for industry, technology and processes, and regulators are each described. The benefits are further categorized.

Figure 3 outlines the benefits of lean authoring for industry, technology and processes, and regulators.

Time savings and efficiency resulting from implementing lean authoring vary. Gains depend on

- the scope of the change
- the templates and digital tools used
- stakeholder acceptance of the changes
- the business process, and
- the training methods.

Benefits realized by the authors’ companies include

- Significant reductions in document length (approximately 50% at Merck [1])
- Faster or less complicated internal reviews
- Fewer resources consumed to create CSRs
- Shorter duration of writing effort for registrational CSRs (an average of 5.25 weeks from availability of data to CSR approval), and
- Less time from availability of data to CSR approval for registrational CSRs (reductions up to 25% at Novartis, 50% at Pfizer, and 90% at Novo Nordisk).

Novo Nordisk’s CSR automation using a lean template requires approximately 35 h of authoring effort per CSR from database lock to CSR approval. Additionally, by centralizing the CSR authoring process to a team of 4 writers who automate all the company’s CSRs, Novo Nordisk significantly improved efficiency, freeing 37 other writers to permanently prioritize other business needs.

## Challenges to Implementing Lean Authoring and Solutions

Even though the benefits of lean authoring are clear, its implementation comes with challenges that can hinder successful adoption. Understanding and addressing these challenges is crucial for organizations aiming to implement or enhance lean authoring practices.

**Fig. 4:**
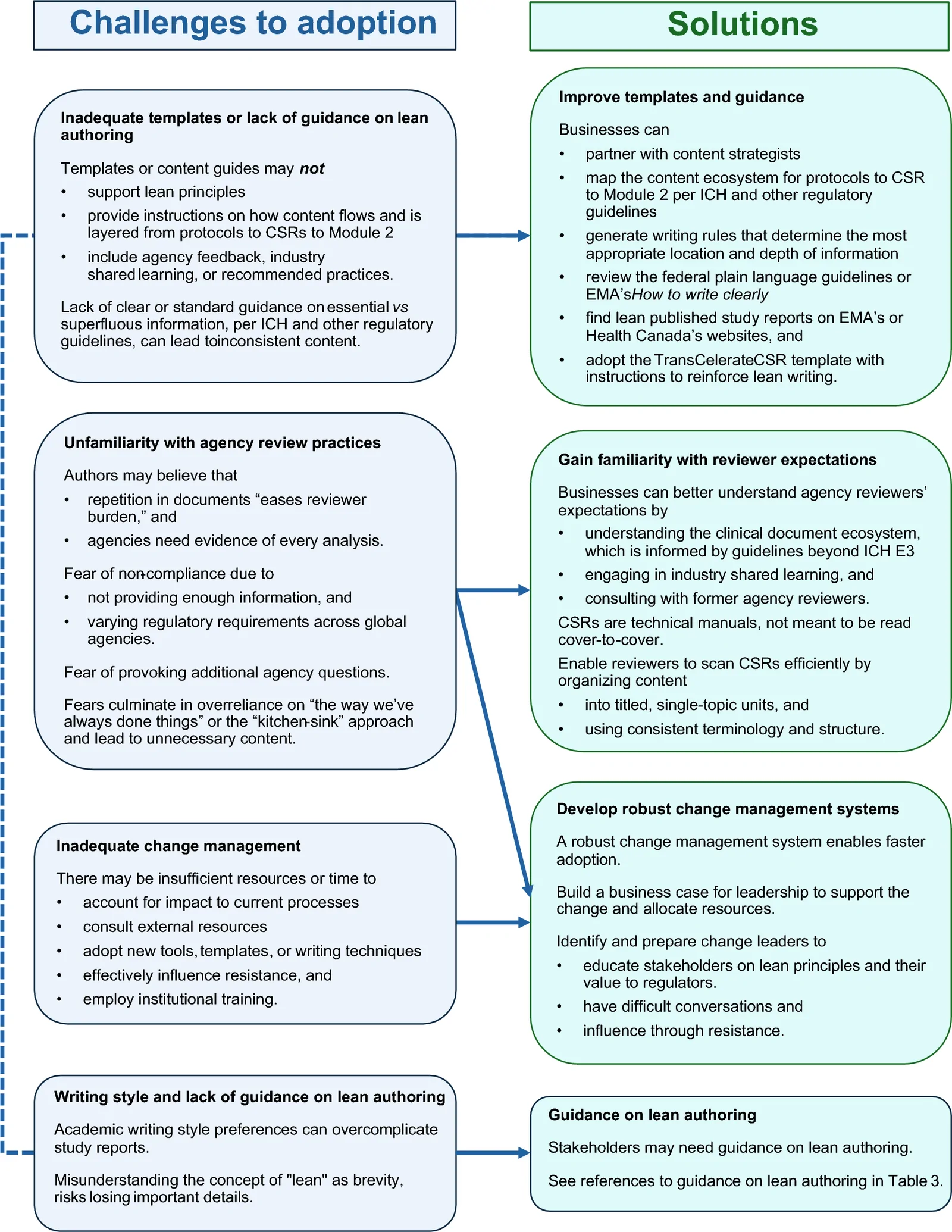
Challenges and solutions to implementing lean authoring. The challenges to adoption of lean authoring are shown alongside solutions to overcome these challenges. Challenges and solutions are further categorized, but solutions may apply to more than one challenge. For example, challenges due to inadequate change management practices are countered by tips to bolster change management and change leaders, but will also be impacted by improving templates, targeting the primary audience and developing robust education for stakeholders.

Figure 4 identifies common challenges to lean authoring adoption that the authors’ organizations have faced, alongside proposed solutions. Note that the solutions may map to more than one challenge.

### Improve Templates and Guidance

Using templates, such as TransCelerate’s, and content guides that support lean writing principles can streamline the writing process. The TransCelerate template, widely used by the industry, allows for leaner text but may be open to interpretation. Clear and consistent template or procedural guidance on essential information, coupled with thorough planning and standardized terminology, can mitigate this pitfall.

Mapping the CSR template or content guide with ICH guidelines in Modules 2 and 5 and creating content rules will clarify where content belongs in the eCTD, if not in the CSR. Collaborating with a consultant who was formerly an agency reviewer can also help map the content to meet regulators’ expectations. These actions will make it easier to address concerns from cross-functional stakeholders. Novo Nordisk’s lean authoring journey started with thorough content mapping. This foundation, along with positive feedback from ex-agency reviewers, is the robust justification from which Novo Nordisk drove change acceptance.

Exchanging experiences among companies using the TransCelerate template can boost its potential. Furthermore, promoting collaboration and sharing recommended practices across companies can help align lean authoring with regulatory standards. This collaboration could lead to the acceptance of the lean CSR as an industry standard.

### Gain Familiarity with Reviewer Expectations

Clinical authoring teams must weigh the relative risk of including too much data versus too little.

Excessive data can confuse regulators and lead to more reviewer questions. Comparatively, lean CSRs include fewer data points or textual remarks to validate and do not include interpretive content that exceeds the scope of the CSR per ICH. Structured authoring further reduces reviewer burden by drawing on established principles of information organization to reduce extraneous processing demands during review [16]. Confusing, overly complicated Module 2 clinical documents that do not adequately explain the key findings may also result in additional question-and-answer cycles, document resubmission, or regulatory requirements post-approval.

For example, Lilly received feedback from FDA reviewers indicating that overly long clinical documents were often skipped or led to confusion. In some cases, this led to delayed or missed opportunities for approval. Industry consultations with current and ex-FDA reviewers have confirmed this practice as being more widespread [6].

Because of the importance of understanding the regulator’s perspective on lean authoring, this topic is explored more in the next section.

### Develop Robust Change Management Systems and Educate Stakeholders on Their Target Audience

Strong change management programs and communication plans are essential to shift internal stakeholder mindsets toward adopting lean authoring.

A robust change management plan is crucial. In 2020, Lilly adopted the TransCelerate CSR template into their quality system, transitioning from a 14-section to an 8-section report. Despite being part of the TransCelerate initiative, reports over 10,000 pages continued.

Key steps in the change management process that could have been improved were

- Socializing the change across functions to share the vision and purpose
- Engaging employees by explaining benefits
- Anticipating shifts by listening to concerns, and
- Reinforcing the change through ongoing education.

Similarly, Sanofi introduced the TransCelerate CSR template in 2018 to limit CSR size, but issues persisted due to overreporting efficacy and safety information to “ease regulator review.” To address this, Sanofi is in the process of preparing a change communication plan with a dedicated change management team to educate writers and stakeholders about regulatory reviewer practices and expectations. They plan to lead a multi-platform communication and education strategy, including surveys, newsletters, webinars, shared learning forums, and video testimonials highlighting success stories.

Consistent with Lilly, Sanofi’s example underscores the importance of

- Anticipating concerns
- Developing robust educational materials, and
- Imparting knowledge of why the benefits of change outweigh the risks.

Roche understood that socialization is important to overcome discomfort with change and took a feed-forward and step-wise approach to introducing lean authoring, prioritizing writer training and stakeholder alignment. Writers were trained on influential leadership, the new methods, and expectations of their role. Stakeholders were engaged through awareness sessions and senior leadership mandates. An ongoing learning forum was created to refine the process.

People are usually resistant to being told, first, what others want them to do. A successful approach to tailoring change messages to cross-functional stakeholders is instead, first, understanding their concerns and motivations and, next, adapting the message about the change as a solution. Influential leaders can bring others along and make them feel that they are part owners of the change, and the change is a solution to their unique problem.

Leading change, then, requires a willingness and proficiency to adapt to difficult conversations. Change leaders must be able to explain the changes’ alignment to ICH and regional guidelines, as well as any available agency feedback or competitive intelligence. Change leaders must understand the analyses with enough technical and scientific proficiency to challenge authoring teams to think critically about the evidence they want to provide in CSRs. This may mean hiring change leaders with a combination of technical, scientific, and influential leadership proficiencies.

To leverage the digital authoring tool to generate CSRs, Novo Nordisk created a new, dedicated role within the writing function. Writers in this new role are content writers who are both highly skilled in managing projects, stakeholders, and change management processes, and who possess technical skills to use the digital tool, manage the digital content library, and educate stakeholders on ICH-driven content mapping rules. Establishing the new role and CSR-focused team cultivated the level of expertise needed to drive adoption of the digital tool, enabling Novo Nordisk to streamline the CSR process quickly and efficiently.

Through training and experience, writers often have greater institutional and individual knowledge of the required content of regulatory documents than other stakeholders. Because writers lead clinical document development, they have a greater responsibility to guide teams on process and content. Writers must be capable of implementing key talking points and may become change leaders. However, this does not obviate the need to partner with experienced change leaders, allies within key functions, and senior leaders who can influence cross-functional partners at every level in the organization.

## Regulator Reactions to Lean (or Not-So-Lean) Authoring

Central to the implementation of lean authoring is answering the question of whether leaner documents will impact regulatory review.

Although global regulators have stated that verbose and repetitive documents delay reviews [6], consistent evidence suggests that agency reviewers favor lean and structured CSRs.

Balanced content in CSRs is crucial, yet no clear guidelines inform the industry on how much information is just enough. Authoring teams must align with regulator expectations to balance the risk of not including enough detail versus including too much.

Ways to obtain regulatory reviewers’ viewpoints on lean CSRs versus verbose or lengthy ones include

- Unsolicited feedback provided by regulators during review
- Solicited feedback collected by process owners, regulatory stakeholders, and writers
- Shared knowledge from other pharmaceutical companies or representatives of participating healthcare regulatory agencies in collaborative industry initiatives, such as TransCelerate
- Partnership with vendors specializing in regulatory document development, and
- Partnership with regulatory policy scientists.

### Input from regulators on clinical study report length, content, and format

#### Solicited and Unsolicited Feedback from Regulators

Soliciting feedback from reviewers at healthcare regulatory agencies can happen through questions in a planned regulatory interaction, questions to the project manager at the agency, or more creative solutions, such as surveys.

One team at Lilly solicited feedback directly from active FDA reviewers to pioneer an ICH-driven content map for eCTD modules 2 and 5. The FDA reviewers were former colleagues of an ex-agency reviewer, who also happened to be a former colleague of a leader in Lilly’s writing function. The reviewers were spread across divisions and were asked in email to indicate preference for choices on data repetition, document format, and length. The reviewers stated a clear preference for lean text and not repeating the same data across text, tables, and figures. They cited quality concerns as well as the burden of conducting their own quality review.

Obviously, lean CSRs do not guarantee regulatory outcomes, and not all CSRs are part of an application. Several confounding factors drive regulatory outcomes independent of document style, including the strength of data, scientific merit, and risk–benefit analysis in the application. However, the Lilly team that pioneered the pilot content map had so much success with their lean documents that FDA and EMA agency reviewers cited the clear articulation of the benefits and risks in the documents as reasons for

- Priority review or no clock stop for questions
- Regulator interest in approval of other indications
- Reversal of previous unfavorable regulator decisions
- Development of a new FDA guidance that borrowed from Lilly’s documents, and
- Granting of market exclusivity.

Preceding Lilly’s adoption of lean authoring, FDA reviewers frequently complained about the length of Lilly’s CSRs. Some pointed to the federal plain language guidelines, while others demonstrably rewrote sections of Lilly’s reports. In several cases, excessively long documents were cited as quality issues or as contributors to an unfavorable review.

Prior to Novo Nordisk’s adoption of lean authoring, they received feedback from regulators on conflicting messages across documents caused by inappropriately placed content.

#### Consultation with Former Agency Reviewers

Examples from Lilly, Novo Nordisk, and Sanofi demonstrate how consulting with former agency reviewers can be beneficial.

Lilly and Novo Nordisk both independently presented former agency reviewers with two CSRs based on the same study: the original CSR written without following lean authoring methods and a lean version. Novo Nordisk took their consultation one step further and generated the lean version of their CSR using their automated reporting tools. Novo Nordisk and Lilly received similar feedback from their consultants. The former regulators preferred the revised, lean CSR.

Sanofi’s consultation with an ex-FDA reviewer allowed them to create lean content rules to guide the interpretation of the TransCelerate template.

Nielsen et al. [10] outlined a list of questions to ask former agency reviewers to align on the right amount of objective content to place in CSRs.

### Publicly Available Regulator Feedback on Clinical Study Report Length, Content, and Format

Professional service vendors can assist in adopting structured authoring, component content libraries, content mapping, and GenAI to create CSRs. Content strategy vendors may be useful to lead the change management process with communication plans and training. Knowing that other companies have successfully implemented lean authoring to create CSRs can help socialize adoption.

In lieu of or in addition to consultation with former agency reviewers and content strategy vendors, companies can consider the publicly available resources listed in Table 3. Many of these can assist in striking the right balance between lean content and what regulators expect.

**Table 3:** Publicly available perspectives from regulators on lean authoring

Resource	Description of utility	Web address
FDA manual of good review practices	Documented “best practices” developed to improve the quality of reviews and review management, provide consistency in review practices, and transparency to the public.	https://www.fda.gov/about-fda/center-drug-evaluation-and-research-cder/cder-manual-policies-procedures-mapp
US federal plain language guidelines	Originated in the mid-1990s by the US federal government to facilitate clear and concise communication across federal agencies. Source material for structured authoring.	https://www.plainlanguage.gov/law/
EMA’s “How to write clearly” publication	Originated to facilitate clear and concise communication across the European Commission. Similar to the US federal plain language guidelines.	https://data.europa.eu/doi/10.2782/022405
ICH E3 (R1)	The ICH E3 and E3 (R1) Q&A documents describe how the ICH guidelines for creating CSRs should be used.	https://www.ich.org/page/efficacy-guidelines
ICH M4E (R2)—clinical summaries ICH E2C (R2)—core safety	Teams that want to include data outside the scope of the CSR can be directed to other locations in the eCTD ecosystem where such data are better suited. In addition, lean authoring principles, such as cross-referencing instead of repeating and keeping text brief (with page limits), are encouraged in the M4E (R2) guidance.	https://www.ich.org/page/ich-guidelines https://www.ich.org/page/multidisciplinary-guidelines
CORE (Clarity and openness in reporting: E3-based) reference	Manual created by American Medical Writers Association (AMWA) and European Medical Writers Association (EMWA) volunteers to help medical writers understand ICH E3 guidelines. It is referenced as one of the sources used in development of the TransCelerate CSR template [28].^1^	https://www.core-reference.org/
TransCelerate templates	TransCelerate is a consortium composed of industry and agency members aimed at harmonizing CSR format and content to reduce complexity, increase quality, and ease review and interpretation by healthcare regulatory agency reviewers, IRBs, and ethics committees.	https://www.transceleratebiopharmainc.com/assets/clinical-content-reuse-solutions/
Value of Medical Writing: The Regulator’s Perspective	Publishes the results of a survey sent to reviewers at 8 global healthcare regulatory agencies. Respondents cited verbosity, document length, and data repetition as the biggest quality issues leading to delays in approval.	Original print: https://amwajournal.org/index.php/amwa/article/view/83/70 EMWA reprint: https://journal.emwa.org/medical-devices/value-of-medical-writing-the-regulators-perspective/

^1^Hamilton et al. suggested a content and formatting guide to adapt the 2018 release of the TransCelerate CSR template. TransCelerate has since updated the template to incorporate feedback from Hamilton et al.’s review, other input and current regulatory practices

## Conclusions

The pharmaceutical industry is increasingly focusing on speed and agility to outpace competitors, capture key markets [25, 26], and reach patients faster. This increases the burden on cross-functional authoring teams to create a higher volume of complex documents more quickly and on healthcare regulatory agency reviewers to consume the documentation.

Lean authoring is a strategic approach that aims to streamline the creation of clinical documents, including CSRs, making them easier for reviewers to navigate and logically follow. Regulatory agency reviewers previously characterized verbosity, lack of clarity, poor rationale, and non-adherence to ICH guidance as document quality issues that they encounter [6]. Lean authoring addresses these concerns through clarity, brevity, and audience targeting, which, in the context of this paper, is based on a thorough mapping of the ICH guidelines to frame the scope and purpose of the CSR relative to other documents in the ecosystem.

Yet many companies are experiencing innovation or change fatigue, especially since the COVID-19 pandemic. Deep-rooted cultures of traditional, academic authoring practices, lack of understanding of regulatory reviewers’ needs, and ineffective change management practices may hinder adoption. Companies can successfully transition to lean authoring by effectively articulating the benefits of lean authoring, obtaining strong support from senior stakeholders, and providing adequate training and change management.

The pharmaceutical landscape is changing rapidly. At the forefront of these changes are automation and AI. Automation aims to increase efficiency, reduce errors, and free up human resources for more strategic tasks. In the context of medical writing, automation can help streamline document creation, data analysis, and other repetitive tasks. In this article, we’ve suggested that content needs to be well-structured and adapted before digitalization to realize the full potential of AI. In other words, “AI is only as good as the content it is trained on.” As Bill Gates summed up, “automation applied to an inefficient operation will only amplify the inefficiency [27].” In a future article, we will explore how AI is changing the field of medical writing and how companies can prepare to fully embrace this powerful new technology for clinical documentation.

While Novo Nordisk and Lilly obtained opinions of former agency reviewers to inform their content strategies, the authors invite regulatory reviewers to comment on the ideas in this paper.

Perhaps collaborative groups, such as TransCelerate, Clinical Trials Transformation Initiative, The Organisation for Professionals in Regulatory Affairs, or others, could work with regulators to promote lean authoring.

## Supplementary Information

Below is the link to the electronic supplementary material.Supplementary file1 (DOCX 25 KB)

## Data Availability

No datasets were generated or analysed during the current study.

## References

[1] Brown E, Jochman K. Regulatory matters. Med Writ. 2021;30(1):69–71.

[2] European Commission: Directorate-General for Translation, Field Z. How to write clearly [Internet]. Publications Office of the European Union. 2015. Available from: 10.2782/022405

[3] Bhardwaj P, Sinha S, Yadav RK. Medical and scientific writing: time to go lean and mean. Perspect Clin Res. 2017;8(3):113–7. 28828305 10.4103/picr.PICR_11_17 PMC5543761

[4] Latino M. Lean Authoring: Bringing Efficiency and Speed to Clinical Study Reports [Internet]. Precision for Medicine. 2024. Available from: https://www.precisionformedicine.com/blog/lean-authoring-bringing-efficiency-and-speed-to-clinical-study-reports

[5] Cuppan G. Interview by author, 10 June 2025. Email transcript.

[6] Cooper J, James LC, Affleck J, et al. Value of medical writing: the regulator’s perspective. AMWA J. 2021;36(4):145–51.

[7] Hofmann H. Search for the real and other essays. In: Hayes ST, Hayes BH Jr, editors. Cambridge, Massachusetts: The M.I.T. Press; 1967.

[8] Carroll JM, van der Meij H. Ten misconceptions about minimalism. IEEE Trans Prof Commun. 1996;39(2):72–86.

[9] Schriver KA. Dynamics in document design: creating text for readers. New York: John Wiley & Sons, Inc; 1996.

[10] Nielsen IO, de Langsdorff V, April J. How Medical Writing and Regulatory Affairs Professionals Can Embrace and Deploy Generative AI at Scale [Internet]. Applied Clinical Trials. 2025. Available from: https://www.appliedclinicaltrialsonline.com/view/medical-writing-regulatory-affairs-professionals-embrace-deploy-generative-ai

[11] International Organization for Standardization . Plain language — Part 1: Governing principles and guidelines (ISO Standard No. 24495–1:2023). 2023.

[12] ASD. ASD-STE100: Simplified Technical English (Issue 9). ASD-STE100 Maintenance Group (STEMG). 2023.

[13] Anderson RD, Eberlein KJ (Eds). Darwin Information Typing Architecture (DITA) Version 1.3 Part 1: Base Edition. 2015. OASIS Standard. Available from: https://docs.oasis-open.org

[14] Cuppan GP, Bernhardt SA. Writing for the Biopharmaceutical Regulatory Reader. 2nd ed. McCulley Cuppan Inc.; 2025. pp. 8–10; 13.

[15] Food and Drug Administration. Guidance for Industry, E3 Structure and Content of Clinical Study Reports, Questions and Answers (R1) Page [Internet]. Available from: https://www.fda.gov/media/84857/download

[16] Yu JS, Yao Y. Structured and intelligent technical writing. In: Intelligent language services. Singapore: Springer; 2026. p. 271–96.

[17] plainlanguage.gov. Plain Language Guidelines Page, Revision 1 [Internet]. 2011. Available from: https://www.plainlanguage.gov/media/FederalPLGuidelines.pdf

[18] Horn RE, Nicol EH, Kleinman JC, et al. Information Mapping for Learning and Reference [Internet]. Information Resources Inc. Cambridge, MA. 1969. Available from: https://apps.dtic.mil/sti/tr/pdf/AD0699201.pdf

[19] Kargren M, April J, Clark G, et al. Unlocking new efficiencies: how structured content authoring is streamlining the production of clinical documents for the pharmaceutical industry. Med Writ. 2023;32(3):32–7.

[20] Krause M, Chang N. Achieving 10,000× training data reduction with high-fidelity labels [Internet]. Google Research Blog; 2025. Available from: https://research.google/blog/achieving-10000x-training-data-reduction-with-high-fidelity-labels/

[21] Food and Drug Administration. FDA Announces Completion of First AI-Assisted Scientific Review Pilot and Aggressive Agency-Wide AI Rollout Timeline [Internet]. 2025. Available from: https://www.fda.gov/news-events/press-announcements/fda-announces-completion-first-ai-assisted-scientific-review-pilot-and-aggressive-agency-wide-ai#:~:text=FDA%20NEWS%20RELEASE-,FDA%20Announces%20Completion%20of%20First%20AI%2DAssisted%20Scientific%20Review%20Pilot%20and%20Aggressive%20Agency%2DWide%20AI%20Rollout%20Timeline,-More%20Press%20Announcements

[22] Food and Drug Administration. FDA Launches Agency-Wide AI Tool to Optimize Performance for the American People [Internet]. 2025. Available from: https://www.fda.gov/news-events/press-announcements/fda-launches-agency-wide-ai-tool-optimize-performance-american-people#:~:text=Built%20within%20a%20high-security,develop%20databases%20for%20nonclinical%20applications

[23] European Medicines Agency. EMA regulatory science to 2025 – strategic reflection [Internet]. 2020. Available from: https://www.ema.europa.eu/en/documents/regulatory-procedural-guideline/ema-regulatory-science-2025-strategic-reflection_en.pdf

[24] European Medicines Agency. NDSG workplan 2025–2028. Data and AI in medicines regulation [Internet]. 2025. Available from: https://www.ema.europa.eu/en/documents/other/network-data-steering-group-workplan-2025-2028_en.pdf

[25] Dukart H, Lanoue L, Rezende M, et al. Emerging from disruption: the future of pharma operations strategy [Internet]. McKinsey & Company. 2022. Available from: https://www.mckinsey.com/capabilities/operations/our-insights/emerging-from-disruption-the-future-of-pharma-operations-strategy

[26] Schwinn L, White N. The New Pharma Playbook: Speed and Efficiency [Internet]. Korn Ferry. 2024. Available from:https://www.kornferry.com/insights/perspectives/the-new-pharma-playbook-speed-and-efficiency

[27] Brandon J. 25 Quotes from Bill Gates on How to Succeed [Internet]. Inc. 2016. Available from https://www.inc.com/john-brandon/25-of-the-best-bill-gates-quotes-on-success.html

[28] Hamilton S, Bernstein AB, Blakey G, et al. Critical review of the TransCelerate template for clinical study reports (CSRs) and publication of Version 2 of the CORE Reference (Clarity and Openness in Reporting: E3-based) Terminology Table. Res Integr Peer Rev. 2019;4:1–9. 10.1186/s41073-019-0075-5. 31406582 PMC6683477

