# Supplementary material for: Optimizing Clinical Study Report Development Through Lean Authoring: A Framework for Meeting Regulatory Expectations and Preparing for Automation
Source: Ther Innov Regul Sci. 2026 Jun 10;60(5):1232–46. doi: 10.1007/s43441-026-00980-6 (PMC13526120; doi:10.1007/s43441-026-00980-6)
Supplement: Supplementary file 1 [file 43441_2026_980_MOESM1_ESM.docx]

**Supplemental Table 1	Differentiating the audience of clinical study reports from other closely related clinical study documents**

| Audience | Scope of interest in clinical study documents |
| --- | --- |
| Healthcare regulatory agency reviewers | **Role:** Responsible for   - ensuring regulatory compliance - reviewing data supporting conduct of the clinical trial, and - reviewing data supporting results   **Need:** *Concise*, high-quality information in reports from clinical studies supported by robust data [6]. |
| Institutional ethics committees / Institutional review boards | **Role:** Responsible for approval of clinical study protocols and informed consent forms.  **Need:** Ethically responsible trial designs described in study protocols that focus on participant safety and benefit to patients. |
| Healthcare professionals / physicians | **Role:** Responsible for patient access to available prescription drugs.  **Need:** Personnel involved in clinical trials require concise and clear clinical study protocols, investigator’s brochures, and publicly available data (data from clinical trials posted on transparency websites, such as clinicaltrials.gov, public assessment reports on EMA’s website and publications) to make ***quick*** benefit / risk decisions. |
| Patients | **Role:** May be presented with *implications* of medical findings from a body of evidence familiar to their physician.  **Need:** Clear and concise lay summaries of clinical data that explain the medical findings. Public audiences have access to redacted study results in EU and US government databases. |
